# Tree–Hillclimb Search: An Efficient and Interpretable Threat Assessment Method for Uncertain Battlefield Environments †

**DOI:** 10.3390/e27090987

**Published:** 2025-09-21

**Authors:** Zuoxin Zeng, Jinye Peng, Qi Feng

**Affiliations:** 1 School of Information Science & Technology, Northwest University, Xi’an 710127, China; pjy@nwu.edu.cn; 2 School of Electronics and Information, Northwestern Polytechnical University, Xi’an 710129, China; fq19990906@mail.nwpu.edu.cn

**Keywords:** Bayesian networks, uncertain battlefield environment, threat assessment, expert knowledge, structure learning, sensitivity analysis

## Abstract

In uncertain battlefield environments, rapid and accurate detection, identification of hostile targets, and assessment of threat levels are crucial for supporting effective decision-making. Despite offering the advantage of structural transparency, traditional analytical methods rely on expert knowledge to construct models and often fail to comprehensively capture the non-linear causal relationships among complex threat factors. In contrast, data-driven methods excel at uncovering patterns in data but suffer from limited interpretability due to their black-box nature. Owing to probabilistic graphical modeling capabilities, Bayesian networks possess unique advantages in threat assessment. However, existing models are either constrained by the limitation of expert experience or suffer from excessively high complexity due to structure learning algorithms, making it difficult to meet the stringent real-time requirements of uncertain battlefield environments. To address these issues, this paper proposes a new method, the Tree–Hillclimb Search method—an efficient and interpretable threat assessment method specifically designed for uncertain battlefield environments. The core of the method is a structure learning algorithm constrained by expert knowledge—the initial network structure constructed from expert knowledge serves as a constraint, enabling the discovery of hidden causal dependencies among variables through structure learning. The model is then refined under these expert knowledge constraints and can effectively balance accuracy and complexity. Sensitivity analysis further validates the consistency between the model structure and the influence degree of threat factors, providing a theoretical basis for formulating hierarchical threat assessment strategies under resource-constrained conditions, which can effectively optimize sensor resource allocation. The Tree–Hillclimb Search method features (1) enhanced interpretability; (2) high predictive accuracy; (3) high efficiency and real-time performance; (4) actual impact on battlefield decision-making; and (5) good generality and broad applicability.

## 1. Introduction

The evolution of modern warfare, especially rapid advancements in aircraft and missile technologies, has profoundly altered the offensive and defensive dynamics on the battlefield, significantly compressing the decision-making window for the defender [1,2]. Under these circumstances, rapid and accurate threat assessment is an unprecedented challenge in uncertain battlefield environments. The ability to efficiently detect, identify, and assess targets—including their state, intent, and threat level—is directly related to the effectiveness of critical decisions such as evasive maneuvers and fire allocation, and it is crucial for enhancing overall operational effectiveness.

Currently, threat assessment methods primarily develop along two paths: analytical methods (linear weighting methods) and data-driven methods (non-linear reasoning methods). Analytical methods, such as the Analytic Hierarchy Process (AHP) [3,4], Multi-Attribute Decision-Making (MADM) theory [5], and TOPSIS [6,7], rely on expert knowledge to construct theoretical models and evaluate threats through using quantitative indicators. Their advantage lies in the simplicity and transparency of the model structure, and they can achieve high accuracy when expert knowledge is reliable and comprehensive. However, in the face of increasingly complex threat factor systems, existing expert knowledge often fails to comprehensively depict intricate, non-linear causal relationships, leading to limited model representational capacity and potentially decreased assessment accuracy. Data-driven methods, such as neural networks [8,9,10,11], support vector machines (SVMs) [12], and Bayesian networks (BNs) [13,14,15], learn non-linear mapping from threat attributes to assessment results directly from data, thereby reducing strong reliance on expert knowledge and excelling at uncovering complex patterns hidden within the data. However, their significant drawback—being treated as “black-box”—makes it difficult to effectively integrate and utilize valuable domain expert knowledge. Furthermore, data-driven methods suffer from poor interpretability and lack transparency in their reasoning processes.

As a key representative of data-driven methods, Bayesian networks (BNs), due to their integration of probability theory and graph theory, demonstrate unique value in the field of threat assessment under uncertain battlefield environments [16,17,18,19]. They can clearly represent the dependencies among threat factors and effectively integrate expert knowledge to handle situations with incomplete information, with their reasoning process being more aligned with human cognitive patterns. There are currently two main approaches for constructing Bayesian networks:

- One is to build the network based on expert knowledge, typically resulting in single-layer or multi-layer naive models. For example, Zhou Yuan proposed a threat assessment model for key point air defense targets based on a Naive Bayesian Network [20]; Tang Shuai proposed a threat assessment model based on a hierarchical Naive Bayesian network according to the indicator system architecture for potential threat assessment of underwater platforms [21].
- The other is to use structure learning algorithms to directly learn the network structure from data. For example, Li Ye proposed a dynamic programming Bayesian network structure learning algorithm integrating MWST and an improved MMPC algorithm and applied it to construct a target threat assessment model [22]; Shang Shanshan proposed a particle swarm optimization algorithm integrating a Salp Swarm Algorithm (SSA) mechanism and modeled submarine threat assessment [23].

However, both of these methods have limitations: models constructed by experts heavily rely on predefined causal relationships, and when experts fail to fully grasp all causal relationships among complex factors, the model structure may become inaccurate, affecting the accuracy of reasoning and assessment precision; while models obtained through purely data-driven structure learning often exhibit excessively high structural complexity, leading to large scoring computations, they significantly increase algorithmic computational time and storage requirements, making it difficult to meet the stringent real-time demands of uncertain battlefield environments.

To address these challenges, this paper aims to explore a novel Bayesian network threat assessment construction paradigm and develop its specific implementation, the Tree–Hillclimb Search method [24]. This method is dedicated to becoming an efficient and interpretable threat assessment solution for uncertain battlefield environments. Its core idea is to integrate expert knowledge as constraints into the structure learning process, seeking a superior balance among model accuracy, complexity, and real-time performance. Specifically, based on expert knowledge, Tree–Hillclimb Search takes the initial network structure as its foundation and constraint, ensuring the model possesses a reasonable logical starting point grounded in domain knowledge and interpretability; it then employs structure learning algorithms to reveal causal dependencies among variables and subsequently conducts guided corrections to the initial network within the constraint framework based on the learning results and sensitivity analysis. This hybrid mechanism allows for flexible adjustment according to the relative reliability of expert knowledge versus data. It is anticipated that this model will integrate the advantages of both approaches:

- First, Tree–Hillclimb Search compensates for localized gaps in expert knowledge through data learning, thus achieving higher precision than expert empirical models.
- Second, by incorporating expert constraints to limit structural complexity, Tree–Hillclimb Search maintains significantly lower model complexity and computational overhead compared to complex networks learned purely from data. This enables effective improvements in evaluation accuracy and speed within acceptable computational costs, better meeting the rapid decision-making demands in uncertain battlefield environments.

This paper conducts research (under the typical uncertain battlefield environment case of naval air defense) to demonstrate the effectiveness and superiority of Tree–Hillclimb Search.

## 2. Task Scenario

The uncertain battlefield environment poses significant challenges to threat assessment, characterized by incomplete information, rapidly changing situations, and severely compressed decision-making windows. In the context of naval air defense, the rapid advancement of aviation and missile technologies has profoundly altered the offense–defense balance. The diverse, dense, and high-speed aerial strike capabilities exert unprecedented pressure on surface ships [25]. In such uncertain battlefield environments, the ability to quickly and accurately assess threat levels and provide reliable bases for critical decisions, such as evasive maneuvers and fire distribution, is directly linked to the enhancement of overall combat effectiveness. This paper takes naval air defense as a case study, aiming to explore threat assessment methods suitable for such uncertain battlefield environments. The core idea and methodology are universal and can be extended to other battlefield scenarios with similar uncertainties.

**Figure 1:**
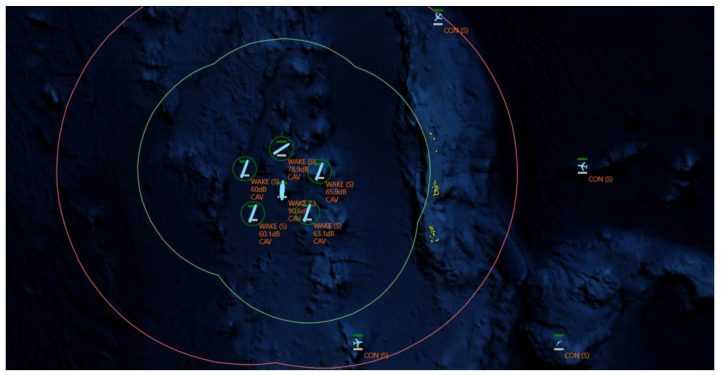
Task scenario diagram of fleet air defense operations.

Therefore, this paper focuses on the air defense operations of our fleet against enemy anti-ship attacks. In the task scenario, our fleet, centered around the flagship, is accompanied by multiple warships equipped with various sensors. The fleet maintains a constant formation during air defense operations. The formation of enemy flight will launch attacks from different directions, approximately 200 km away from the center of our fleet, consisting of two groups of carrier-based attack aircraft, one group of electronic warfare aircraft, and one group of unmanned reconnaissance aircraft, as shown in Figure 1.

According to the task scenario, the dataset used in this paper includes 15 nodes: threat level (Threaten), target type (Type), Target State (State), Time of Arrival (Time), electromagnetic radiation (Detected), Radiation Sources (Sources), electromagnetic detection time (Duration), Altitude, Sensors, time interval (Interval), Heading, Latitude, Longitude, Speed, and Distance. Among these, the parameters of the Threaten, Type, State, and Time nodes cannot be directly obtained or measured by our ships in real-world situations, while the parameters of the other nodes can be measured by our shipborne detection sensors.

Since Bayesian network algorithms are generally not suitable for continuous variables and there are multiple continuous variables in this dataset, along with some discrete variables with a large number of states, it is necessary to discretize certain data nodes before performing Bayesian network structure learning and sensitivity analysis. The state spaces of each node variable are shown in Table 1.

The dataset was generated via a high-fidelity naval combat simulation platform, modeling various encounter scenarios between a fleet and incoming aerial targets. The dataset comprises over thirty thousand data samples, each of which represents a battlefield snapshot containing sensor observations and the corresponding threat level. Table 2 below is a representative example of a data instance:

## 3. Threat Assessment Model Based on Expert Experience

**Figure 2:**
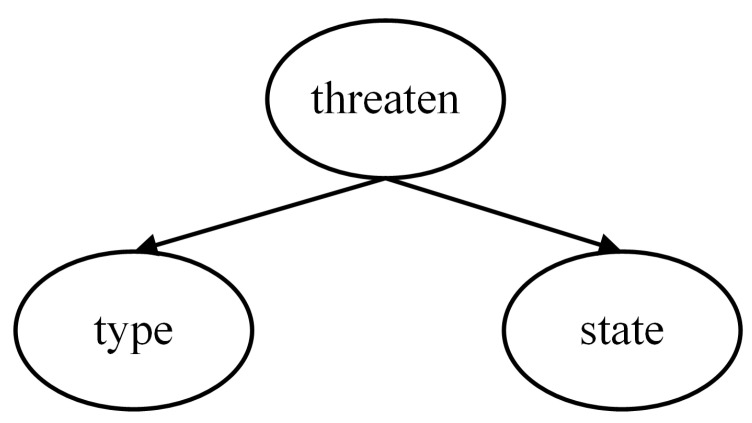
Threat level assessment model.

**Figure 3:**
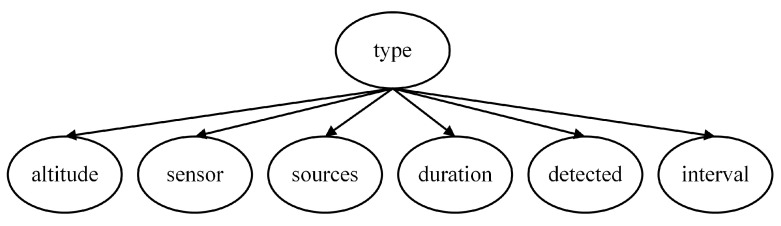
Target type assessment model.

**Figure 4:**
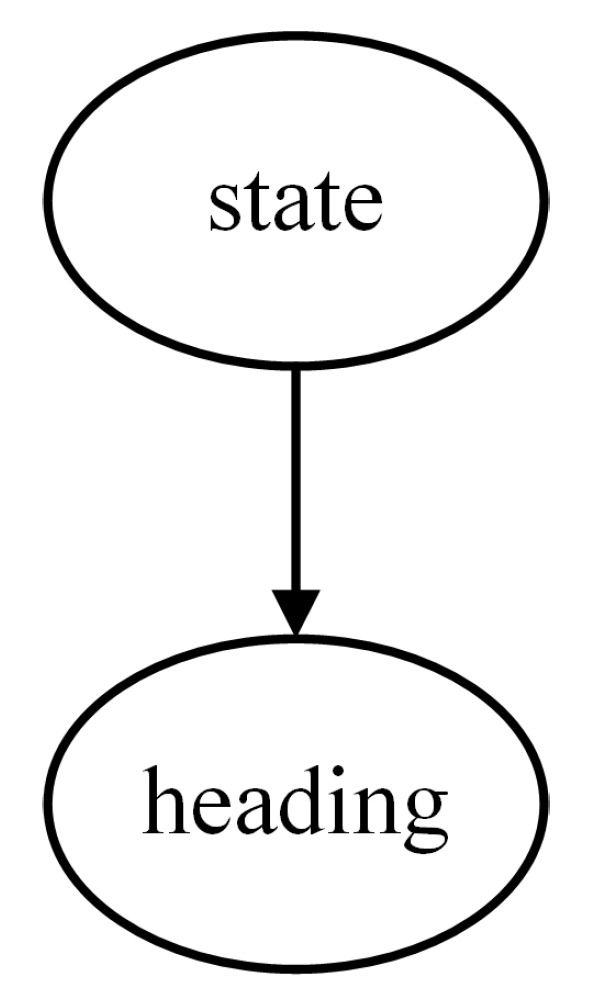
Target State assessment model.

**Figure 5:**
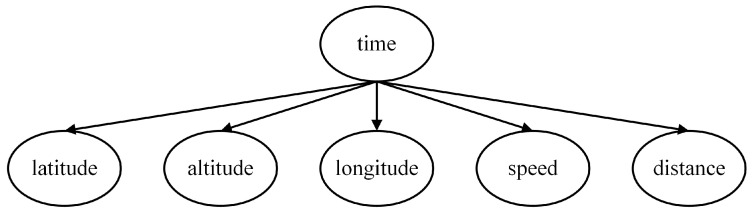
Time to arrival assessment model.

According to the task scenario, the threat level is determined by the target information, which can be expressed using a Naive Bayes model. To enhance the interpretability of the model, several intermediate nodes are introduced. Therefore, a multi-layer Naive Bayes network is employed in this paper to construct the threat assessment model based on expert experience, comprising four local assessment models as shown in Figure 2, Figure 3, Figure 4 and Figure 5.

The threat level serves as the root node of the first layer and the entire network. When evaluating the degree of threat, the focus is on the Type and State of the target. Since these two characteristics directly determine the threat, the feature nodes of the Threaten in the threat assessment model consist solely of the Type and the State, as depicted in Figure 2.

In determining the assessment model of target type, the most intuitive feature node is whether the target is detected (Detected), as this scenario represents the highest likelihood of a particular aircraft type performing its designated mission. Additionally, the duration of electromagnetic detection time (Duration) and the estimated source of electromagnetic emission (Sources) are crucial for identifying the target type. Furthermore, our infrared detection source (Sensor), detection interval (Interval), and target altitude (Altitude) also serve as bases for judging the Type, and thus they are included as child nodes of the Type, as depicted in Figure 3.

For the Bayesian assessment model of the Target State, the root node is the State, and its feature node is the Heading, as depicted in Figure 4.

In addition to the three aforementioned local models, an additional feature node, time to arrival (Time), can be introduced at the lowest level. This node is related to various current motion state parameters of the enemy target. The parameters of the target that can be measured by our warship’s sensors include Latitude, Longitude, Speed, Altitude, and Distance, all of which are incorporated as feature nodes of the Time, as depicted in Figure 5.

**Figure 6:**
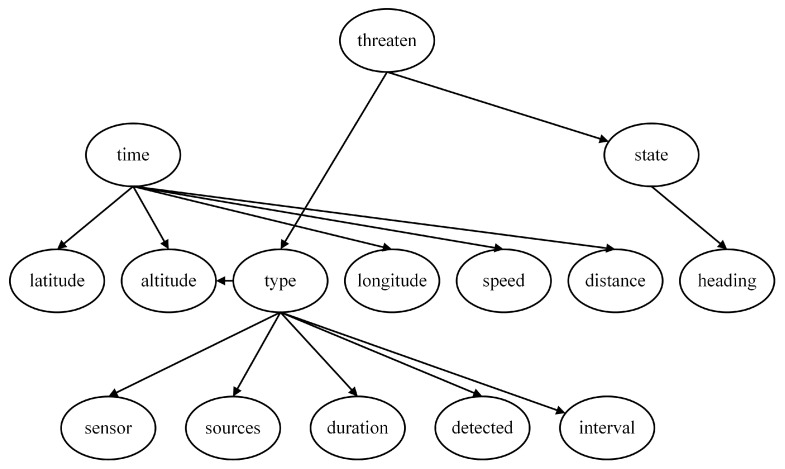
Threat assessment model based on expert experience (NB Model).

By connecting the four local assessment models, a complete threat assessment model based on expert experience is obtained, as depicted in Figure 6. The eleven nodes, including Latitude, Longitude, Speed, Altitude, Distance, Heading, Duration, Detected, Sources, Sensors, and Interval, represent the most basic feature nodes. They constitute the parameters of the unknown aerial target that can be directly acquired or measured by our warship.

## 4. Threat Assessment Model Based on Structure Learning Algorithms

Bayesian network structure learning (BNSL) refers to the process of using certain reasoning algorithms to identify the network structure that best fits the sample dataset without prior knowledge of the network structure or its parameters. It also reveals the qualitative relationships between variables [26,27].

In this study, we select a diverse set of structure learning algorithms as benchmarks to thoroughly evaluate the proposed Tree–Hillclimb Search method against state-of-the-art approaches from different philosophical families. This selection is made to be compared with our method:

- Constraint-based methods: These are represented by the PC-estimate algorithm, which is grounded in statistical conditional independence tests. This approach is theoretically well-founded for learning causal structures and is particularly suitable for revealing the underlying dependency skeleton among variables, making it a robust baseline for comparison.
- Score-based local search methods: These are represented by the Hillclimbing Search algorithm. This approach is chosen for its simplicity, computational efficiency, and widespread adoption in practical applications. It exemplifies the classic trade-off between search efficiency and the risk of converging to local optima.
- Global optimization strategies: These are represented by the Tree Search algorithm. The method of this category aims to search the model space in more detail. Compared with the Hillclimbing algorithm, it is not easy to fall into local optimization, but the computational cost is high.

In the hyper-parameter settings for the simulation tests, the Bayesian Information Criterion (BIC) was adopted as the scoring function for all score-based algorithms (Hillclimbing, Tree Search, and Tree–Hillclimb) due to its desirable property of penalizing model complexity. The search process terminates when no score improvement is observed over 100 consecutive iterations.

This multi-faceted benchmarking approach ensures a comprehensive and fair evaluation of our proposed method against strong and diverse competitors.

### 4.1. PC-Estimate Algorithm

The PC-estimate algorithm is a significant variant of the classical PC algorithm, specifically designed for learning Bayesian network structures from observational data. Its core theoretical foundation lies in conditional independence testing, which assumes that the true network structure encodes the conditional independence relationships among variables [28,29]. The algorithm systematically eliminates non-existent edges through statistical testing, ultimately deriving the network’s skeleton structure (undirected graph) and partially determining the direction of edges, forming a complete partial directed acyclic graph (CPDAG) representing Markov equivalence classes. The pseudocode for the PC-estimate algorithm is presented in Algorithm 1.
**Algorithm 1 **PC-estimate algorithm [30]**INPUT: Vertex set V, Conditional Independence Information****OUTPUT: Estimated skeleton C, separation sets S (only needed when directing the skeleton afterwards)**1. Form the complete undirected graph *C* on the vertex set *V*.2. l=−1; *C* = $\tilde{C}$3. repeat4.      *l* = *l* + 15.      repeat6.              Select a (new) ordered pair of nodes i,j that are adjacent such that              |adj(C,i)∖{j}|≥l7.              repeat8.                    Choose (new) k⊆adj(C,i)∖{j} with |k|=l9.                    if *i* and *j* are conditionally independent given *k* then10.                          Delete edge i,j11.                          Denote this new graph by *C*12.                          Save *k* in S(i,j) and S(j,i)13.                    end if14.              until edge i,j is deleted or all k⊆adj(C,i)∖{j} with |k|=l have been chosen15.        until all ordered pairs of adjacent variables *i* and *j* such that |adj(C,i)∖{j}|≥l           and k⊆adj(C,i)∖{j} with |k|=l have been tested for conditional independence16. until for each ordered pair of adjacent nodes i,j:|adj(C,i)∖{j}|<l

The algorithm starts with a complete undirected graph, assuming potential connections between all variables. Its core phase employs a stratified conditional independence testing strategy, beginning with zeroth-order conditioning (unconditional independence testing) and progressively increasing the number of conditioning variables (k=0,1,2,…). At each layer, the algorithm tests the independence of adjacent variable pairs X−Y given a candidate conditioning set *Z* of size *k*. If there exists a *Z* that renders *X* and *Y* conditionally independent, the connecting edge is removed, and *Z* is recorded as the separating set. This process iterates until all adjacent variable pairs have been tested or the conditioning set size reaches a preset limit. Unlike the traditional PC algorithm, PC-estimate introduces a stability enhancement mechanism: it generates candidate skeletons through multiple data subsamples and filters edges based on their frequency of appearance in the subsamples, retaining only those with stability exceeding a threshold τ. This design aims to improve robustness in scenarios with small samples and high dimensions. After the skeleton is determined, the algorithm identifies V-structures (triplets of the form X→Z←Y) using Sepset information and applies the Meek ruleset to propagate directional constraints, ultimately outputting a CPDAG.

**Figure 7:**
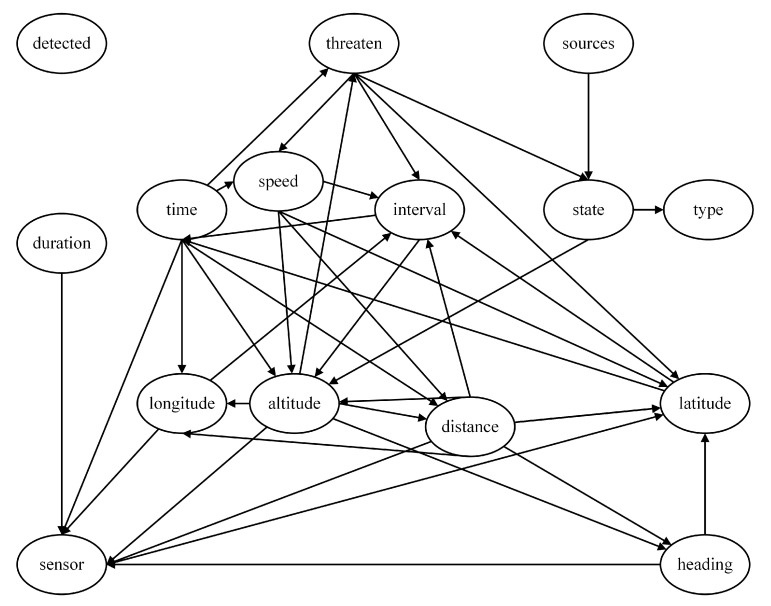
Threat assessment model based on PC-estimate algorithm (PC-estimate model).

Using the PC-estimate algorithm for Bayesian network structure learning, the resulting threat assessment model is shown in Figure 7, referred to as the PC-estimate model. In this model, the Detected node has no connecting edges to other nodes, and a directed cycle “Time→Speed→Interval→Time” appears, which is not allowed in Bayesian networks. Consequently, the PC-estimate model is unreasonable and unsuitable for further work; it should be discarded.

### 4.2. PC-skeleton_to_pdag Algorithm

The PC-skeleton_to_pdag algorithm is a core directional module within the classical PC structure learning framework, responsible for converting an undirected skeleton graph into a partially directed acyclic graph (PDAG) representing Markov equivalence classes. Its design objective is to maximize the determination of edge directions while maintaining structural equivalence consistency, under the premise of satisfying d-separation semantics. The pseudocode for the PC-skeleton_to_pdag algorithm is presented in Algorithm 2.
**Algorithm 2 **PC-skeleton_to_pdag algorithm [30]**INPUT: Skeleton $G_{\mathrm{skel}}$, separation sets S****OUTPUT: CPDAG G**1. for all pairs of nonadjacent variables i,j with common neighbor *k* do2.        if k∉S(i,j) then3.              Replace i−k−j in $G_{skel}$ i→k←j4.        end if5. end for6. In the resulting PDAG, try to orient as many undirected edges as possible by repeated   application of the following three rules:7. **R1** Orient j−k into j→k whenever there is an arrow i→j such that *i* and *k* are nonadjacent.8. **R2** Orient i−j into i→j whenever there is a chain i→k→j9. **R3** Orient i−j into i→j whenever there are two chains i−k→j and i−l→j such that *k* and *l* are nonadjacent.10. **R4** Orient i−j into i→j whenever there are two chains i−k→l and k−l→j such that *k* and *l* are nonadjacent.

The algorithm takes the skeleton graph and separating sets as input and realizes direction derivation through a two-phase process. In the first phase, V-structures (collider nodes) are identified: for every undirected triplet X-Z-Y (where X and Y are nonadjacent) in the skeleton, if Z is not in the Sepset (X,Y), then Z is inferred to be a collider node, and the direction is set as X→Z←Y. This step is based on the logic of conditional independence—if X and Y are related (non-independent) given Z, then Z must be a common effect of X and Y. In the second phase, the Meek ruleset is applied to propagate directional constraints: based on the partially determined edge directions, the remaining edge directions are iteratively derived using four types of deterministic rules (avoiding new V-structures, preventing cycle formation, transitive orientation, and repelling conflicting directions). For example, if there exists A→B—C and A and C are nonadjacent, then B→C is mandatorily oriented to avoid generating an unobserved V-structure A→B←C; if there exists A→B→C and A—C, then A→C is oriented to prevent cycle formation. This process continues until no new edges can be oriented, outputting a PDAG containing both directed and undirected edges, where undirected edges represent uncertain dependency relationships within the equivalence class.

**Figure 8:**
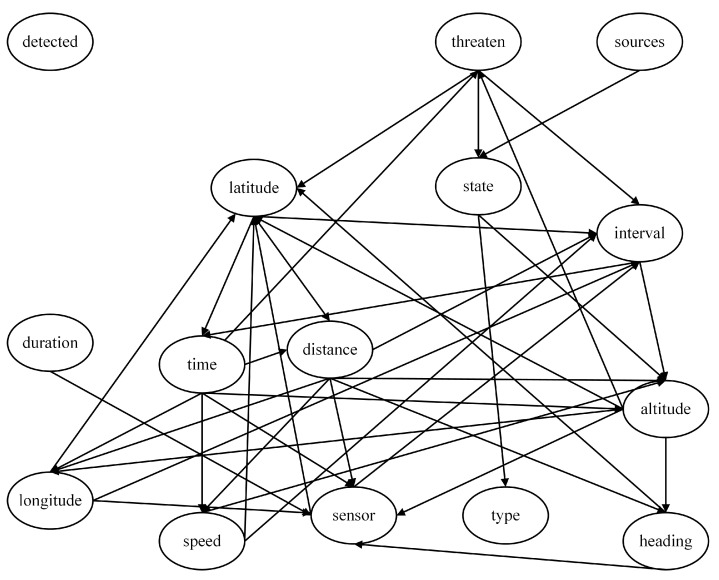
Threat assessment model based on PC-skeleton_to_pdag algorithm (PC-skeleton_to_pdag model).

Using the PC-estimate algorithm for Bayesian network structure learning, the resulting threat assessment model is shown in Figure 8, referred to as the PC-skeleton_to_pdag model. In this model, the Detected node has no connecting edges to other nodes, and a directed cycle “Latitude→Time→Speed→Latitude” appears, which is not allowed in Bayesian networks. Consequently, the PC-skeleton_to_pdag model is unreasonable and unsuitable for further work; it should be discarded.

### 4.3. Hillclimbing Search Algorithm

The Hillclimbing Search algorithm is a classic local optimization method within the framework of Bayesian network structure learning based on scoring search. The core idea is to transform the structure learning into a combinatorial optimization problem: defining a scoring function to quantify the fitness of the network structure to the data and using heuristic search to find the graph model with the best score [31,32]. Due to its simplicity and computational efficiency, this algorithm has become a widely adopted basic method in practical applications. The pseudocode for the Hillclimbing algorithm is presented in Algorithm 3.
**Algorithm 3 **Hillclimbing Search algorithm**INPUT: Variables set X, dataset D, scoring function f****OUTPUT: Bayesian network G**1. Initial network structure *G*2. bestScore←f(G∣D)3. while true4.        NewScore←−∞,NewDAG←null5.        for each do obtained by the add-edge, delete-edge, and reverse-edge operators $G^{\prime }$6.                $currentScore\leftarrow f\left(G^{\prime }|D\right)$7.                if currentScore>newScore8.                        $newScore\leftarrow currentScore,newDAG\leftarrow G^{\prime }$9.                end if10.          end for11.      if newScore>bestScore12.                bestScore←newScore,G←newDAG13.        else14.                return *G*15.        end if16. end while

Hillclimbing Search starts with a randomly generated or empty network as the initial structure and iteratively executes the optimization loop of local perturbation, evaluation, and selection. In each iteration, the algorithm generates a set of neighboring structures for the current network, usually including three types of operations: adding valid edges (without introducing cycles), deleting existing edges, or reversing edge directions (while maintaining acyclicity). Each candidate structure is evaluated by a scoring function (such as the Bayesian Information Criterion (BIC), the Bayesian Dirichlet equivalent (BDe) score, or the Minimum Description Length (MDL)), and the structure with the greatest score improvement is selected as the starting point for the next round of search. This process continues until convergence conditions are met (such as no improvement in the neighborhood or reaching the maximum number of iterations). To avoid local optimum traps, strategies such as random restarts (randomly initializing new starting points) or taboo lists (recording recent operations to avoid cycles) are often employed. The final output is the directed acyclic graph (DAG) with the highest score.

**Figure 9:**
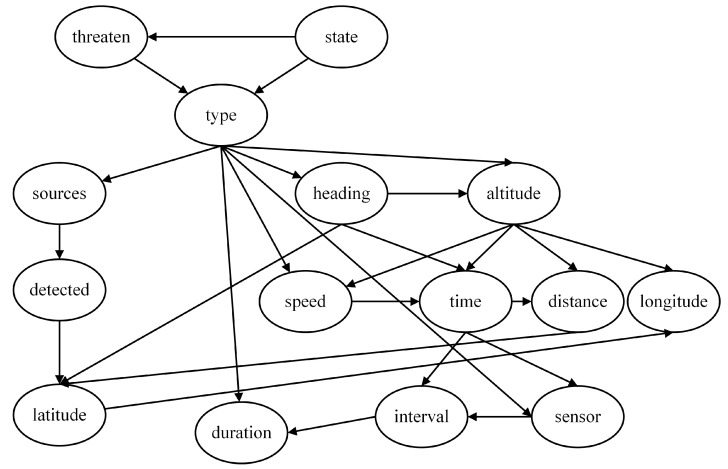
Threat assessment model based on Hillclimbing Search algorithm (Hillclimbing Search model).

Using the Hillclimbing Search algorithm for Bayesian network structure learning, the threat assessment model obtained, as shown in Figure 9, is called the Hillclimbing Search model. This model includes all nodes, and the network structure does not contain directed cycles, indicating that the Hillclimbing Search model is reasonable and suitable for subsequent work.

### 4.4. Tree Search Algorithm

The Tree Search algorithm is a class of Bayesian network structure learning methods based on systematic space enumeration, whose core idea is to model the network structure learning as an optimal path search problem in the state space [33,34]. Unlike local optimization strategies, this algorithm explicitly constructs a search tree to traverse the possible network structure space, aiming to balance computational feasibility and global optimality, especially suitable for precise learning of medium-sized networks. The pseudocode for the Tree Search algorithm is presented in Algorithm 4.
**Algorithm 4 **Tree Search algorithm [35]**INPUT: Mutual information ${\left\{I\left(i,j\right)\right\}}_{i\neq j}$****OUTPUT: Edge set E**1. E:={}2. ε:={{i,j}∣i≠j}3. ε:=ε∖{{i,j}} for {i,j}∈ε maximizing I(i,j)4. if (V,E∪{{i,j}}) does not contain a loop, then E:=E∪{{i,j}}5. if ε≠{}, then go to 3, else terminate( ∪ and ∖ denote the addition and subtraction of two sets)

The algorithm starts with an empty graph or a preset initial structure as the root node and iteratively extends child nodes to generate a search tree. Each node represents a partial or complete network structure, and edges represent structural transformation operations (such as adding, deleting, or reversing directed edges). The search process relies on heuristic scoring functions (such as BIC or BDeu) to evaluate the fitness of structures and employs branch-and-bound strategies for space pruning: setting a score threshold for the current best solution and discarding branches whose upper bound score is below the threshold (proving that they cannot yield a better solution). Typical implementations include two paradigms:

- Breadth-First Search (BFS): Expanding all possible operations layer by layer, using score bounds for pruning to ensure the global optimal solution is found within a limited depth;
- A* Search: Introducing a heuristic function to estimate the potential score gain of unexplored paths, prioritizing the expansion of the most promising branches to improve search efficiency.

The termination condition is usually set as the completion of the search tree traversal or reaching the computational resource limit, with the output being the DAG structure corresponding to the historically best score.

**Figure 10:**
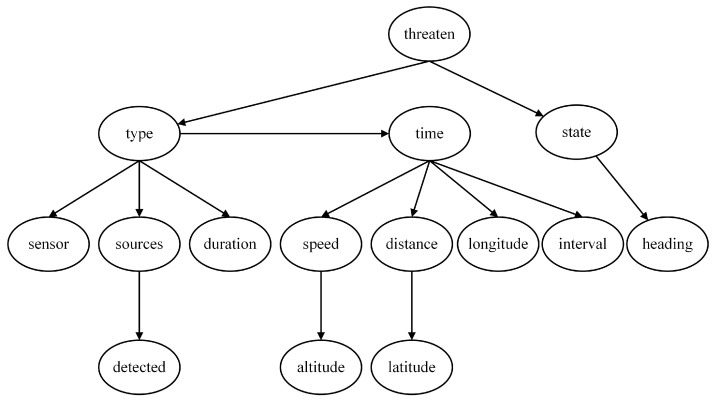
Threat assessment model based on Tree Search algorithm (Tree Search model).

Using the Tree Search algorithm for Bayesian network structure learning, the threat assessment model obtained, as shown in Figure 10, is called the Tree Search model. This model includes all nodes, and the network structure does not contain directed cycles, indicating that the Tree Search model is reasonable and suitable for subsequent work.

### 4.5. TAN Model

The Tree–Augmented Naive Bayes (TAN) model is a semi-naive classifier based on the Bayesian network, proposed by Nir Friedman et al. in 1997 [36]. While retaining the efficiency of the Naive Bayes model, the TAN model introduces a tree-like conditional dependence structure among attribute variables, significantly improving classification performance and maintaining computational efficiency. This model balances complexity and expressiveness, making it particularly suitable for complex scenarios with significant correlations among attributes.

**Figure 11:**
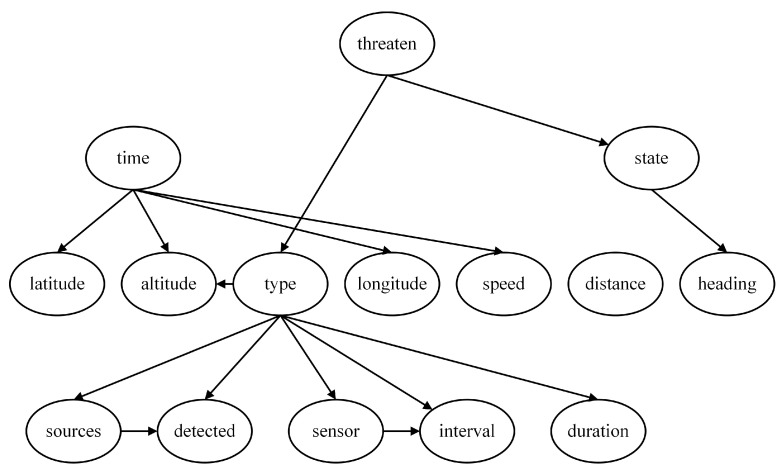
Tree-Augmented Naive Bayes model for naval air defense threat assessment (TAN model).

By examining the Hillclimbing Search model and the Tree Search model, we can observe that although their dependency and independence relationships are not entirely the same, some dependency relationships are reflected in both models, such as “Sources→Detected”, “Sensor→Interval”, and “Interval→Duration”. Based on this understanding, these edges are added to the expert experience model to establish a TAN model, as shown in Figure 11, serving as the fourth network structure for subsequent sensitivity analysis work.

## 5. Sensitivity Analysis of Threat Assessment Models

### 5.1. Sensitivity Analysis

The sensitivity analysis of Bayesian networks focuses on quantifying the impact of local parameter changes on global inference conclusions, thereby revealing the intrinsic dependency relationships among system variables [37,38,39]. Bayesian networks naturally depict causal dependencies among variables through directed acyclic graphs and utilize conditional independence to efficiently decompose high-dimensional joint probability distributions, significantly reducing the modeling and computational burden of complex systems. This characteristic not only enhances the model’s applicability to multi-attribute complex scenarios but also simplifies the implementation of sensitivity analysis—by systematically perturbing the conditional probability parameters of specific nodes, the propagation effect on the target query can be precisely traced without the need for repeated global computations. This method quantifies parameter sensitivity, identifies the sources of uncertainty that have the greatest impact on outputs, and provides a theoretical basis for optimizing data collection and improving model robustness.

The choice of sensitivity metrics is critical for accurately characterizing the influence of threat factors on the assessment outcome. Rather than relying on a single measure, we employ a trio of complementary metrics—variance reduction ($V_{r}$), mutual information (MI), and expected variance ($V_{E}$)—to provide a holistic view. Each metric illuminates a different aspect of “influence”:

- $V_{r}$ quantifies the extent to which knowing a feature’s value reduces the uncertainty (variance) in the threat level. It answers the following question: “How much does a feature reduce the uncertainty of the target variable?”
- MI measures the amount of information shared between a feature and the threat level, capturing all forms of statistical dependence, both linear and non-linear. It answers the following: “How much does knowing this feature tell me about the threat level?”
- $V_{E}$ captures the degree to which changes in a feature cause shifts in the expected value (mean) of the threat level. It answers the following: “Does this feature systematically increase or decrease the estimated threat score?”

This multi-faceted approach is necessary because a feature might be highly informative (high MI) without drastically changing the mean threat estimate (low $V_{E}$), or vice versa. Using all three metrics mitigates the potential bias of any single measure and provides a more robust foundation for formulating resource allocation strategies.

### 5.2. Sensitivity Analysis Based on Variance Reduction

Variance reduction is an indicator that measures the impact of a feature on the uncertainty of the target variable [40]. When the value of a node (feature) is known, the conditional variance of the target variable decreases. The greater the reduction in variance, the greater the impact of the feature on the target variable, indicating stronger sensitivity; conversely, weaker sensitivity is indicated. The calculation formula for variance reduction is shown in Equation (1):

(1) $$V_{r}\left(F\right)=V\left(Q\right)-E\left[V\left(Q|F\right)\right]$$

where *Q* is the target variable, which in this paper refers to Threaten; V(Q) represents the unconditional variance of the target variable; *F* denotes the node to be analyzed, referring in this paper to target parameters such as Speed and Distance; and E[V(Q∣F)] is the expectation of the conditional variance, whose calculation formulas are shown in Equations (2) and (3):

(2) $$V\left(Q\right)=\sum_{q}P\left(q\right)\cdot {\left(X_{q}-E\left[Q\right]\right)}^{2}$$

(3) $$E\left[V\left(Q|F\right)\right]=\sum_{f}P\left(f\right)\cdot V\left(Q|f\right)$$

where *Q* is the target variable, $X_{q}$ is the numerical mapping of each state of the target variable, $E\left[Q\right]=\sum P\left(q\right)\cdot X_{q}$ is the unconditional expectation, *F* is the node to be analyzed, $V\left(Q|f\right)=\sum_{q}P\left(q|f\right)\cdot {\left(X_{q}-E\left[Q|f\right]\right)}^{2}$ is the conditional variance given F=f, and $E\left(Q|f\right)=\sum_{q}P\left(q|f\right)\cdot X_{q}$ is the conditional expectation.

The calculation of the sensitivity of each node to the Threaten node is shown in Table 3. $V_{r}\in \left[0,V\left(Q\right)\right]$, and a higher value indicates a stronger impact of the feature on the uncertainty of the target variable. Considering the sensitivity analysis results of each threat assessment model, a sensitivity ranking based on variance reduction is provided: Distance, Heading, Speed, Latitude, Longitude, Sensor, Altitude, Interval, Sources, Duration, and Detected.

### 5.3. Sensitivity Analysis Based on Mutual Information

Mutual information measures the statistical dependence between a node and the target variable, reflecting the amount of information that a feature provides about the target variable [41]. In sensitivity analysis, it represents the amount of information shared between a feature and the target variable. The greater the mutual information, the more information the node provides about the target node, indicating a stronger correlation between the feature and the target variable, and thus the greater importance of the feature in predicting the target variable.

The definition of mutual information is based on the joint probability distribution P(X,Y) and the marginal probability distributions P(X) and P(Y), as shown in Equation (4):

(4) $$I\left(F;Q\right)=\sum_{f}\sum_{q}P\left(f,q\right)\cdot \log_{2}\left(\frac{P(f,q)}{P(f)\cdot P(q)}\right)$$

where *Q* is the target variable, which in this paper refers to Threaten; *F* denotes the node to be analyzed, referring in this paper to target parameters such as Speed and Distance; P(f) and P(q) are the marginal probabilities of *F* and *Q*, respectively; and P(f,q) is the joint probability of *F* and *Q*. The logarithm used in mutual information I(F;Q) is based on 2 as the base, and the unit is bits.

The calculation of the sensitivity of each node to the Threaten node is shown in Table 4. I(F;Q)≥0, and a higher value indicates that the feature provides more information about the target variable. Considering the sensitivity analysis results of each threat assessment model, a sensitivity ranking based on mutual information is provided: Distance, Speed, Heading, Sensor, Longitude, Sources, Duration, Detected, Altitude, Latitude, and Interval.

### 5.4. Sensitivity Analysis Based on Expected Variance

The expected variance measures the degree of change in the conditional expectation E[Y∣X] of the target variable *Y* as the input variable *X* changes, indicating the average impact on the expected value *Y* of *X*. The core idea is to calculate the conditional expectations of the target variable given different states of each feature node and then compute the variance of these conditional expectations [42], as defined in Equation (5):

(5) $$V_{E}=\mathrm{Var}\left(E\left[Y|X\right]\right)$$

where E[Y∣X] is the conditional expectation of *Y* given *X*. When the input variable *X* assume different states $X_{i}$, the expected value of the target variable *Y* changes, and the expected variance quantifies the extent of this change, as shown in Equation (6):

(6) $$V_{E}=E\left[{\left(E\left[Y|X\right]-E\left[E\left[Y|X\right]\right]\right)}^{2}\right]$$

If *X* has a strong influence on *Y*, then changing the value of *X* will significantly alter the expected value of *Y*, leading to large differences in E[Y∣X] across different values of *X* and consequently resulting in a large variance $V_{E}$. Conversely, if *X* has no influence on *Y*, then E[Y∣X] remains nearly constant, and the variance $V_{E}$ is approximately zero.

Based on the expected variance indicator, the sensitivity of each node to the Threaten node is calculated, with the results presented in Table 5. $V_{E}\in \left[0,V\left(Q\right)\right]$, and a higher value indicates a greater impact of the feature on the conditional expectation of the target variable. Considering the sensitivity analysis results of each threat assessment model, a sensitivity ranking based on expected variance is provided: Sources, Heading, Distance, Speed, Longitude, Sensor, Latitude, Altitude, Interval, Detected, and Duration.

### 5.5. Comparative Visualization of Sensitivity Results

**Figure 12:**
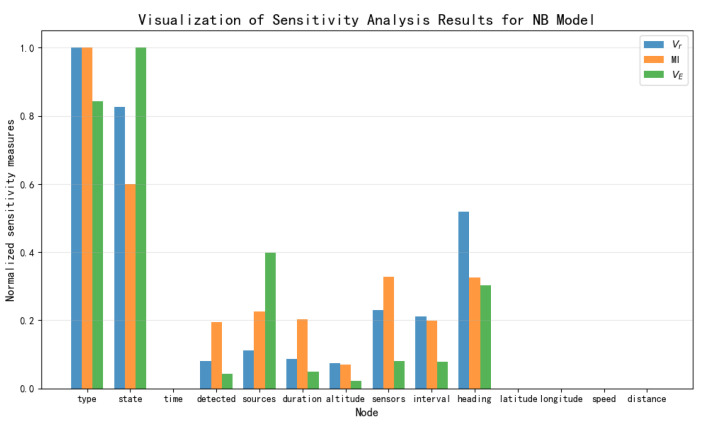
Visualization of sensitivity analysis results for NB Model.

**Figure 13:**
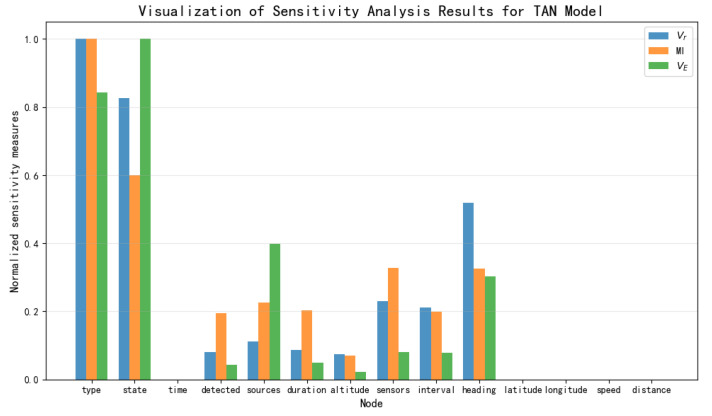
Visualization of sensitivity analysis results for TAN model.

**Figure 14:**
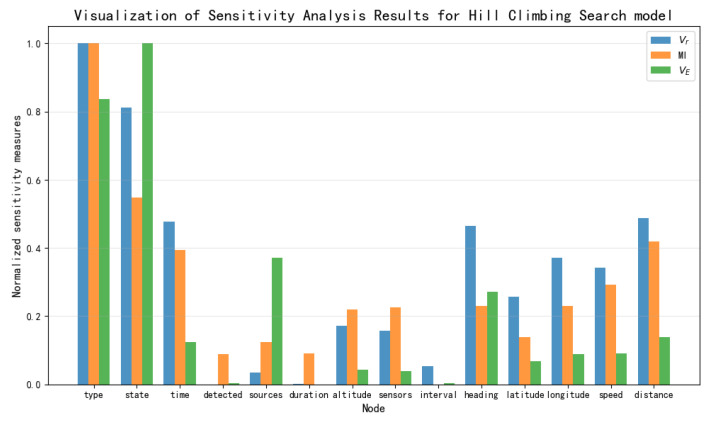
Visualization of sensitivity analysis results for Hillclimbing Search model.

**Figure 15:**
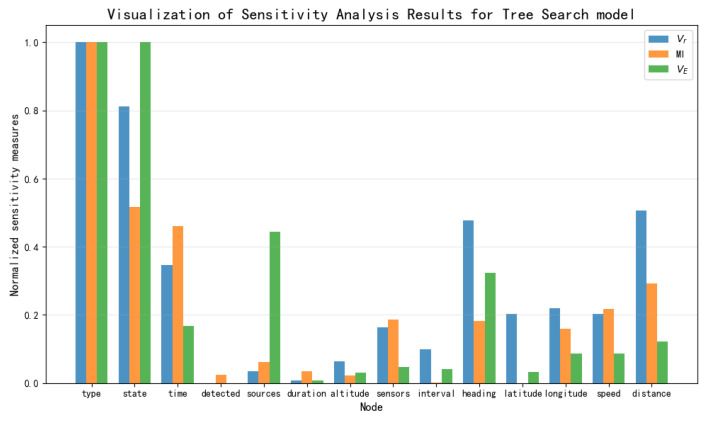
Visualization of sensitivity analysis results for Tree Search model.

Based on the data presented in Table 3, Table 4 and Table 5, the results of the sensitivity analysis were visualized as shown in Figure 12, Figure 13, Figure 14 and Figure 15. The three sensitivity metrics—$V_{r}$, MI, and $V_{E}$—for the NB Model, TAN model, Hillclimbing Search model, and Tree Search model were each normalized via min-max scaling to the range [0, 1]. A separate visualization of the sensitivity analysis results was generated for each model. These visualizations facilitate a direct comparison of how each metric ranks the importance of key threat factors.

By comprehensively considering the above sensitivity ranking, the degree of influence of the 11 threat elements directly obtainable in this paper’s naval air defense threat assessment model on threat assessment is determined, yielding the following sensitivity ranking: Heading, Distance, Speed, Sensor, Latitude, Longitude, Interval, Altitude, Sources, Duration, and Detected. Among these, Heading and Distance have a strong influence on threat assessment, followed by Speed and Sensor, while Latitude and Longitude still have a certain degree of influence. Nodes such as Interval, Altitude, Sources, Duration, and Detected are considered to have minimal impact.

## 6. Tree–Hillclimb Search: Construction and Validation of an Efficient and Interpretable Threat Assessment Method

### 6.1. Model Construction

This section delineates the construction process of the core method proposed in the title—Tree–Hillclimb Search. The method aims to build a threat assessment model for uncertain battlefield environments that balances efficiency (low complexity, rapid inference) with interpretability. Its cornerstone is a structure learning mechanism grounded in expert knowledge constraints: an initial network structure constructed from expert knowledge serves as the foundation and constraint framework, mining potential causal dependencies between variables from data using a structural learning algorithm. Guided by the results of this learning and sensitivity analysis, the initial network is then refined within the boundaries set by expert experience. This approach is made to merge the interpretability of expert knowledge with the high-precision potential of data-driven methods, while strictly limiting model complexity through a constraint mechanism, thereby laying the groundwork for rapid inference in uncertain battlefield environments.

The expert knowledge is integrated through a set of hard constraints, denoted as *C*, which rigorously governs the structure learning process. These constraints are formally defined as follows:

- C_preserve (Mandatory Edge Preservation): All directed edges (Xi→Xj) that exist in the initial expert-derived graph G_expert must be preserved. The Algorithm 5 is prohibited from removing or reversing these edges. This directly incorporates expert experience and guarantees that the final model remains interpretable to them.
- C_add (Edge Addition Permission): A new directed edge (Xi→Xj) not in G_expert may be added only if it satisfies two conditions:
  (1) it does not create a directed cycle (C_acyclic);
  (2) the relationship between Xi and Xj is deemed semantically plausible by domain knowledge. This excludes nonsensical connections (e.g., Latitude→Sensor) and focuses the search on meaningful causal pathways (e.g., Speed→Time).
- C_max_parents (Complexity Control): The number of parent nodes |π(Xj)| for any variable Xj must not exceed a predefined maximum *k*. In this study, *k* = 3. Formally, $\forall X_{j}\in V,\;\left|\pi \left(X_{j}\right)\right|\leq k$. By limiting the number of parents, it prevents overfitting and ensures that the resulting network is sparse and computationally efficient, which is paramount for real-time inference.
- C_acyclic (Acyclicity): The graph must remain a directed acyclic graph (DAG) at all times. This is a fundamental constraint of Bayesian networks.

**Algorithm 5 **The Tree–Hillclimb Search algorithm**INPUT: Variables set V; dataset D; expert-derived DAG G_expert; scoring function f; constraint set C.****OUTPUT: Final Bayesian network G_final.**1. Initialize: G_current←G_expert; best_score← f(G_current∣D).2. Define Constraint Set *C*:      • C_preserve: All edges in G_expert must be preserved.      • C_add: New edges (X→Y) are allowed only if they do not form a cycle and X,Y are conceptually related.      • C_max_parents: Max parents for any node ≤k (e.g., *k* = 3).      • C_acyclic: All graphs must be acyclic.3. while not converged do4.   candidates ← *∅*      Generate candidate neighbors by applying operators that comply with constraint set *C*:      • Add-edge: For any allowed edge (X→Y) not in G_current, if Add(X→Y) complies with C_add, C_max_parents, and C_acyclic, add G_candidate = G_current∪ (X→Y) to candidates.      • Reverse-edge: For an existing edge (X→Y) in G_current, if (X→Y) is not in C_preserve, and if Reverse(X→Y) (i.e., converting it to (Y→X)) complies with C_acyclic and C_max_parents for both nodes, add the new graph to candidates.      • Delete-edge: This operator is disabled for any edge in C_preserve. For an edge (X→Y) not in C_preserve, Delete(X→Y) is always allowed and the resulting graph is added to candidates.5.        for each G_candidate in candidates do6.                current_score← f(G_candidate | *D*)7.                if current_score>best_score then8.                        best_score←current_score9.                        G_current←G_candidate10.                end if11.        end for12. end while13. G_final←G_current14. return G_final

The pseudocode above formalizes the integration of expert knowledge through a set of hard constraints *C*. The algorithm begins by initializing the search with the expert-derived structure G_expert (Step 1), ensuring that the process starts from a logically transparent and interpretable baseline. The constraint set *C* (Step 2) is the core mechanism that governs the entire search. The GenerateNeighbors function (Step 4) is now explicitly designed to operate within this constrained solution space. It only generates candidate structures that strictly adhere to all the rules in C. For example, it will never propose to delete an edge protected by the C_preserve. This ensures that the Tree–Hillclimb Search optimizes the model’s fit to the data without compromising the foundational knowledge provided by experts. Therefore, the final output model synergistically combines the interpretability of expert knowledge with the accuracy of data-driven learning, meeting operational requirements in uncertain battlefield environments.

In the context of naval air defense threat assessment, this paper establishes expert constraints based on domain knowledge, such as the following:

- Time is a crucial factor affecting Threaten, with shorter times typically indicating higher threats. Consequently, while adhering to expert knowledge constraints (i.e., retaining Type and State as feature nodes for Threaten), the Time node is also included as a child of Threaten, forming an assessment structure of Threaten → [Type, State, Time].
  This structure is better aligned with the rapidly changing dynamics of the battlefield conditions.
- A focused revision of the dependency relationships for the Time node in the initial expert model is undertaken. Based on kinematic formulas v=s/t and the dependencies revealed by the structure learning model, it is clear that distance (Distance) and speed (Speed) are the most direct and significant factors determining Time. Within the expert knowledge constraint framework, Distance and Speed are thus established as child nodes of Time (Time → [Distance, Speed]). Additionally, an examination of the structure learning outcomes indicates that latitude (Latitude) and longitude (Longitude) primarily influence Time indirectly through their association with Distance, while altitude (Altitude) affects Time through its connection with Speed. Hence, within the same constraint framework, Latitude and Longitude are designated as child nodes of Distance (Distance → [Latitude, Longitude]), and Altitude as a child node of Speed (Speed → Altitude). This revision not only is consistent with geographic spatial relationships and kinematic common sense but also incorporates the specific dependency strengths revealed by the data.

**Figure 16:**
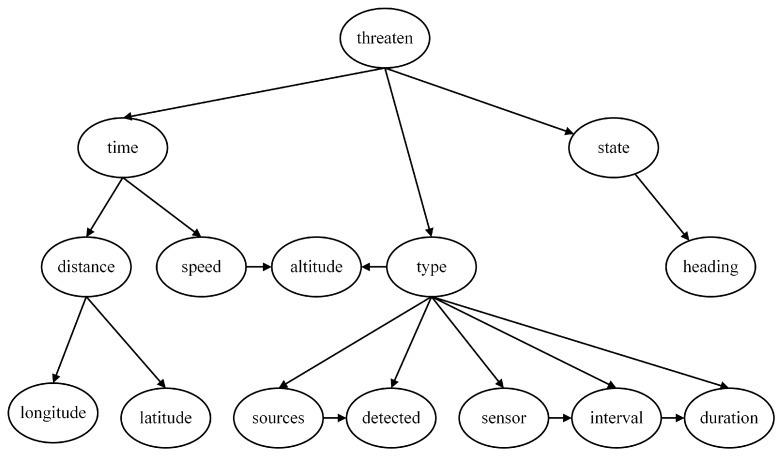
Tree–Hillclimb Search model for naval air defense threat assessment.

Ultimately, through this process of “structure learning based on expert knowledge constraints,” the Tree–Hillclimb Search model is obtained, as depicted in Figure 16. The key advantage of this model is its ability to retain and enhance the interpretable skeleton of the expert model while organically integrating critical causal dependencies revealed by data learning, thereby enhancing the model’s representational capability and expected accuracy. Crucially, the refinements made under constraint guidance keep the model’s complexity at a low level, significantly below that of pure data learning models. This streamlined structure directly translates into a marked improvement in computational efficiency, enabling rapid parameter learning and real-time inference, perfectly suiting the demanding efficiency requirements of uncertain battlefield environments. With the completion of the core model of Tree–Hillclimb Search as an efficient and interpretable threat assessment method for uncertain battlefield environments, its performance will be comprehensively evaluated in subsequent sections.

### 6.2. Evaluation of Model Explainability

Explainability refers to the ability of artificial intelligence systems to make their decision-making logic understandable, trustworthy, and verifiable by human users. This concept goes beyond mere “result explanation” and aims to build a cognitive bridge for human–machine collaboration, focusing on reducing the opacity of machine decisions to align with human cognitive needs and social ethical norms.

Bayesian networks, as a type of probabilistic graphical model, derive their core explainability from the fusion of structural semantic transparency and traceable probability inference. The directed acyclic graph structure of the network explicitly encodes conditional dependency relationships between variables, aligning with human cognitive modular thinking and allowing non-technical personnel to intuitively understand the system’s association mechanism. The Conditional Probability Table quantifies the interaction strength between variables, with prior probabilities representing the base State and conditional probabilities revealing the impact of interventions, thus enabling parameter-supported natural language explanations.

The Tree–Hillclimb Search model is constructed by starting from an expert knowledge model and then incorporating causal dependency relationships among threat elements obtained through structure learning. In addition to retaining the causal dependency relationships of the expert knowledge model, it also integrates deep causal dependencies mined from the data by the structure learning algorithm, rendering the causal relationships between nodes clearer and enhancing the model’s explainability compared to the expert knowledge model.

### 6.3. Simulation Testing Hardware Configuration

All timing experiments were conducted on a standardized hardware and software platform to ensure reproducible results. The specifications are as follows:

- Hardware: The simulation equipment is a ROG Strix 7 Plus purchased in Xi’an, China, with the following hardware specifications: AMD Ryzen 9 7845HX (AMD, Santa Clara, CA, USA) with Radeon Graphics, 16 GB RAM.
- Software: Windows 11 Operating System; Python 3.12.
- Libraries: The pgmpy (v1.0.0) library was used for Bayesian network learning and inference.

All reported times are the average of 1000 independent runs. For inference time, each run involved performing belief propagation with a random set of evidence assigned to the observable nodes. This large number of iterations ensures that the measured times are statistically robust and account for potential runtime variability.

### 6.4. Evaluation of Model Prediction Accuracy

To evaluate the prediction accuracy of different models, we conducted conditional probability reasoning under a specific but representative threat scenario. The scenario is defined as follows: our other sensors (Sensors = 4) detect the electromagnetic radiation (Detected = 1) of an enemy aerial target with moderate altitude (Altitude = 2), small longitude (Longitude = 0), moderate latitude (Latitude = 2), and small heading angle (Heading = 0), but they fail to determine the source of the radiation (Sources = 0), with continuous electromagnetic detection (Duration = 0) and short interval time (Interval = 0). The aerial target is fast (Speed = 4) and at a moderate distance from us (Distance = 2). Analyzing the dataset reveals that, in this scenario, the threat level of the enemy aerial target to us is “high threat” (Threaten = 1).

For the evaluation of model prediction accuracy, this paper employs scenario-based reasoning of the conditional probabilities of the threat level node. To facilitate comparison, the reasoning results of the other four threat assessment models are presented side by side, as shown in Table 6.

The observed marginal difference in accuracy between the proposed Tree–Hillclimb Search model (99.7%) and the unconstrained Hillclimbing model (99.9%) warrants a detailed analysis, as it lies at the heart of the trade-off our method manages. From the perspective of model complexity and overfitting, the Hillclimbing model’s superior accuracy is achieved through a highly complex structure. This complexity grants it a high capacity to fit the training data, but it also inherently increases the risk of overfitting to noise and idiosyncrasies within the specific dataset, potentially compromising its performance and reliability on novel, unseen data. Our model, constrained to a sparser structure, inherently regularizes itself, seeking a more robust and generalizable solution. The negligible 0.2% accuracy difference suggests that the vast majority of the important predictive signals have been successfully captured without resorting to excessive complexity. From the perspective of structural constraints, the expert rules (C_max_parents, C_max_parents) deliberately limit the algorithm’s search space. This prevents it from adding edges that, while potentially yielding a minuscule gain in training accuracy, might be statistically flimsy, causally dubious, or detrimental to model interpretability. The constraints act as a form of domain-knowledge-based regularization, guiding the model toward a solution that is not only accurate but also physically meaningful and trustworthy for human operators. Therefore, this minimal accuracy gap is a conscious and advantageous sacrifice. It is the cost of achieving a model that is five times simpler and ten times faster, and one whose reasoning process can be understood and validated by experts. In the operational context of uncertain battlefields, where rapid decisions based on trustworthy information are paramount, this balance is vastly superior to a marginally more accurate but computationally expensive Hillclimbing Search model.

### 6.5. Evaluation of Model Complexity

In terms of model complexity, this paper first evaluates the parameter complexity of the five threat assessment models, as shown in Table 7. Parameter complexity refers to the size of the Conditional Probability Table required to store the network, which is related to the number of states of the node and the number of parent nodes. From the table, it is evident that the Tree–Hillclimb Search model exhibits significantly reduced complexity compared to the Hillclimbing Search model, greatly diminishing computational requirements. Although its complexity is slightly higher than other models, its accuracy compensates for this drawback.

The processing time of Bayesian networks is closely related to model complexity. Structure learning time measures the time required for the algorithm to discover the causal relationship network among variables from the data, reflecting the algorithm’s ability to effectively capture complex relationships between threat factors and the computational resource requirements of the training process. Parameter learning time measures the time required to estimate Conditional Probability Distributions (CPDs) under a given network structure, reflecting the efficiency in handling different variable state combinations. Inference time measures the time required to calculate the probability distribution of the target variable under given evidence conditions, reflecting the system’s ability to process real-time data and determining the real-time strength of the system’s threat level assessment.

The comparison of inference time is a crucial evaluation metric, which is different from the sensitivity analysis that informed the model design. Sensitivity analysis identifies which features are most influential on the threat level. However, the inference time is determined by the final model’s structural complexity (e.g., number of parameters or connectivity) and dictates the model’s practical viability in a real-time system. A model that is accurate but slow cannot be deployed for time-critical decision-making. Therefore, our model achieves ultra-fast inference while maintaining high accuracy, being strong evidence of its applicability in uncertain battlefield environments. This enables decision-making within seconds in combat scenarios.

As depicted in Table 8, the processing times of the Tree–Hillclimb Search model, including structure learning time, parameter learning time, and inference time, are significantly lower than those of the Hillclimbing Search model and the Tree Search model, demonstrating its superiority in computational resource requirements. Compared to other models, the processing time difference is not substantial, but the Tree–Hillclimb Search model boasts higher accuracy, granting it an advantage.

It should be noted that for the Tree–Hillclimb Search method, the model construction starts from a predefined expert model (Section 3) and undergoes refinement based on subsequent constraints. Importantly, the effort invested by domain experts in building the initial expert experience model constitutes a separate one-time cost and is not included in the structural learning time metrics.

These data fully validate the outstanding performance of the Tree–Hillclimb Search method in terms of “efficiency”. Although the unconstrained climbing search model achieved the highest accuracy (99.9%), its network structure is highly complex, computationally intensive, and prone to overfitting. In contrast, the Tree–Hillclimb Search method not only delivers a substantial 0.6% improvement in accuracy over the pure expert model (Naive Bayes), with nearly identical model complexity and inference speed, but also validates its outstanding efficiency. By successfully constructing a lightweight model through expert knowledge constraints, it achieves ultra-fast real-time inference capabilities—effectively addressing the challenges of rapidly changing information and extremely short decision windows in uncertain battlefield environments. More importantly, the marginal 0.2% accuracy gap between our method and the top performer is a trade-off for a fivefold reduction in model size and a tenfold increase in inference speed. This demonstrates that our constraint-based approach extracts meaningful patterns from data without resorting to bloated structures, thereby achieving overall excellence across all design objectives. Ultimately, the Tree–Hillclimb Search method occupies the unique “sweet spot”, simultaneously satisfying accuracy and efficiency, where others excel in one at the expense of others.

### 6.6. Threat Assessment Strategy

**Figure 17:**
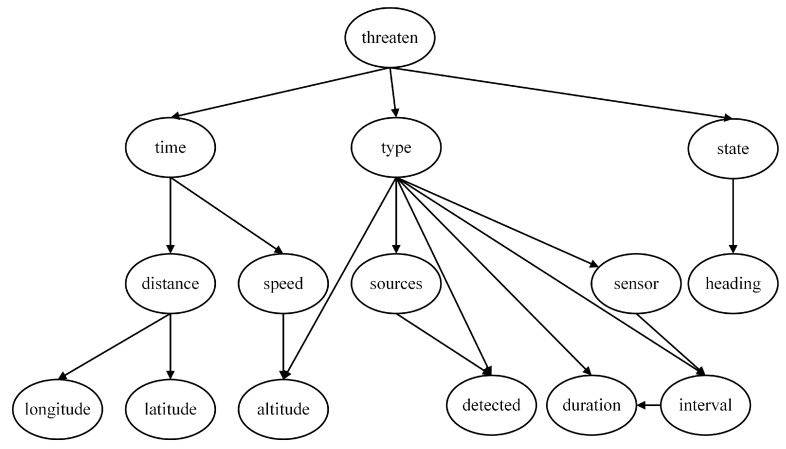
Hierarchical representation of Tree–Hillclimb Search model.

The Tree–Hillclimb Search model is represented in a hierarchical manner, with the Threaten node placed at the top level, its feature nodes Time, Type, and State at the second level, and so on. This hierarchical representation of the Tree–Hillclimb Search model is shown in Figure 17. Comparing this with the sensitivity analysis results, it is evident that nodes at higher levels exhibit higher sensitivity, while nodes at lower levels exhibit lower sensitivity, providing a direct manifestation of the model’s “explainability” advantage. Consequently, a threat assessment strategy, which ensures the detection accuracy of upper-level nodes while relaxing the accuracy requirements for lower-level nodes, can be formulated under such limited-detection-resources constraints. This approach not only guarantees the precision and reliability of threat assessment but also allows for a more rational allocation of sensor resources.

In real-world scenarios, the capability of our surface ships to acquire target information during air defense operations may be subject to various constraints. For instance, limited detection resources may preclude simultaneous high-precision detection of all threat elements, necessitating adjustments to the accuracy requirements for each threat element to accommodate detection resource limitations. Therefore, a reasonable threat assessment strategy is essential to reducing resource consumption while ensuring the reliability and accuracy of threat assessment results.

Through sensitivity analysis, we ascertain the impact of each threat element on threat assessment. Naturally, nodes with higher sensitivity require higher precision, while nodes with lower sensitivity can tolerate lower precision. Hence, a threat assessment strategy can be devised where Heading and Distance are subject to high-precision requirements to minimize misjudgments, while the precision requirements for Speed and Sensor can be slightly relaxed. Furthermore, the precision requirements for Latitude and Longitude can be relaxed even further, and nodes such as Interval, Altitude, Sources, Duration, and Detected can have broader precision requirements, although they should not be entirely neglected. By implementing this strategy under constrained conditions, the reliability and accuracy of threat assessment can be maintained, enabling its more effective application in uncertain adversarial scenarios.

### 6.7. Simulation Validation of Threat Assessment Strategy

Based on the sensitivity ranking results and the proposed threat assessment strategy, the precision of Distance was relaxed from being equally divided into five categories with equal frequency to being equally divided into three categories with equal frequency. The conditional probability of the threat level node was then reasoned under the given scenario, which was consistent with the scenario described in Section 6.6. The reasoning results are presented in Table 9.

Similarly, based on the sensitivity ranking results and the threat assessment strategy provided, the precision of Altitude was relaxed from being equally divided into five categories with equal frequency to being equally divided into three categories with equal frequency. The conditional probability of the threat level node was reasoned again under the given scenario, which was consistent with the scenario described in Section 6.3. The reasoning results are presented in Table 10.

### 6.8. Ablation Study on Expert Constraints

To quantitatively evaluate the contribution of the expert constraints in the Tree–Hillclimb Search method, we conducted an ablation study and compared the performance of three models (Table 11):

1. The Tree–Hillclimb Search model incorporates comprehensive expert constraints and a complete structural learning process;
2. The Hillclimbing Search model operates without expert constraints, initiating structural learning from an empty set with no prior knowledge or constraints;
3. The expert experience model derives its network structure entirely from expert knowledge, without involving any structural learning process.

The results demonstrate a critical trade-off. The unconstrained Hillclimbing model, while achieving the highest accuracy, results in an excessively complex network (2040 parameter complexity), which is over 5 times larger than our proposed model. This complexity directly leads to a 10× increase in inference time (0.0010 s), rendering it impractical for real-time applications. In contrast, our Tree–Hillclimb Search method, guided by expert constraints, successfully identifies a structure that is both highly accurate (a 0.6 improvement over the expert model) and efficient. It maintains a low parameter count and ultrafast inference speed equivalent to the simple expert model. This proves that the expert constraints effectively prevent the algorithm from overfitting to the data and learning spurious, complex relationships, thereby ensuring the model remains interpretable and deployable in resource-constrained, time-sensitive scenarios.

## 7. Conclusions

This paper addresses the requirements of real-time, accuracy, and interpretability in threat assessment under uncertain battlefield environments by proposing and investigating the Tree–Hillclimb Search method—an efficient and interpretable threat assessment approach, which was designed to overcome the limitations of traditional expert models with insufficient representation capabilities as well as pure data-driven models with high complexity and slow computation. The core innovation of this method lies in the implementation of structure learning based on expert experience constraints. The initial network based on expert experience serves as the foundation for constraints, and structural learning algorithms are employed to reveal the causal dependencies among variables hidden in the data. The network is then modified under the guidance of these constraints and sensitivity analysis based on the learning results.

Model evaluation demonstrates that the Tree–Hillclimb Search method effectively combines the advantages of expert experience and data-driven approaches, achieving its design objectives:

- **Enhanced Explainability**: This model preserves the interpretable core framework of expert models while optimizing it with data-driven insights. It uncovers deeper causal relationships, results in a clear and transparent structure, and aligns logically with domain knowledge. This makes its reasoning process highly credible and easily understandable to human operators.
- **High Predictive Accuracy**: The method achieves a 99.7% accuracy in threat assessment, coming within 0.2% of the top-performing unconstrained model while significantly outperforming models such as Naive Bayes, Tree-Augmented Naive Bayes, and Tree Search—effectively handling the uncertainty inherent in battlefield environments.
- **High Efficiency and Real-Time Performance**: Through the strict enforcement of expert constraints, the model complexity is reduced by 80% (403 vs. 2040 parameter complexity) compared to the unconstrained Hillclimbing search, which significantly lowers storage and computational requirements. This reduction directly enables an order-of-magnitude faster inference speed of 0.1 milliseconds—10 times quicker than the unconstrained model—delivering extremely fast real-time reasoning that fully meets the stringent performance demands of uncertain battlefield environments.
- **Actual Impact on Battlefield Decision-Making**: The performance gains of the Tree–Hillclimb Search method have a direct and profound impact on operational effectiveness. Its hierarchical structure aligns closely with sensitivity analysis results, providing reliable guidance for resource optimization strategies—an effect confirmed through simulation validation. The combination of high accuracy and ultra-low latency compresses the OODA (Observe, Orient, Decide, Act) loop, enabling millisecond-level decision-making processes that previously required seconds. This provides commanders with a critical time advantage for executing evasive maneuvers or weapon deployment against high-speed threats. Furthermore, the model’s explainability builds operator trust and supports informed decision-making under pressure.
- **Good Generality and Broad Applicability**: The method is suitable for uncertain battlefield environments and nominal datasets, demonstrating effective threat assessment in scenarios involving uncertain incoming targets based on parameters such as direction and speed. However, the impact of target quantity on threat assessment remains unvalidated. The framework exhibits high transferability to other domains requiring real-time, interpretable, and data-integrated decision-making, such as ground combat, cybersecurity, and civilian applications like traffic management or industrial fault detection. Its core innovation—knowledge-constrained structure learning—is a universal paradigm adaptable to diverse scenarios.

In summary, the Tree–Hillclimb Search method successfully constructs a threat assessment tool that combines high interpretability, near-optimal predictive accuracy, low complexity, and excellent computational efficiency. By leveraging structure learning based on expert experience constraints, it effectively addresses the challenges faced by traditional methods in uncertain battlefield environments, particularly meeting the critical needs of real-time decision-making through its fast reasoning capability and resource optimization strategies. This approach provides a practical and superior solution for real-time threat assessment under uncertainty.

## Figures and Tables

**Table 1:** State spaces of each node variable.

Node	State Spaces and Labels
Threat Level (Threaten)	3: “Low threat”; 2: “Medium threat”; 1: “High threat”; 0: “False target”
Target Type (Type)	5: “Missile”; 4: “Non-stealth carrier-based attack aircraft”; 3: “Electronic warfare aircraft”; 2: “Unmanned aerial vehicle”; 1: “Stealth carrier-based attack aircraft”; 0: “False target”
Target State (State)	2: “Non-RTB”; 1: “RTB”; 0: “False target”
Time of Arrival (Time)	4: “Long”; 3: “Relatively long”; 2: “Medium”; 1: “Shorter”; 0: “Short”
Electromagnetic Radiation (Detected)	1: “Electromagnetic radiation detected”; 0: “No electromagnetic radiation detected”
Radiation Sources (Sources)	2: “Aircraft-mounted active phased array radar”; 1: “Other”; 0: “No radiation detected”
Electromagnetic Detection Time (Duration)	2: “Long detection time”; 1: “Short detection time”; 0: “No electromagnetic radiation detected”
Altitude	4: “High”; 3: “Relatively high”; 2: “Medium”; 1: “Lower”; 0: “Low”
Sensors	4: “Other”; 3: “Active phased array radar”; 2: “Air search radar”; 1: “Electronic warfare equipment”; 0: “Electronic support/measurement system”
Time Interval (Interval)	2: “Long”; 1: “Medium”; 0: “Short”
Heading	2: “Large”; 1: “Medium”; 0: “Small”
Latitude	4: “Large”; 3: “Relatively large”; 2: “Medium”; 1: “Smaller”; 0: “Low”
Longitude	4: “High”; 3: “Relatively high”; 2: “Medium”; 1: “Lower”; 0: “Small”
Speed	4: “Fast”; 3: “Relatively fast”; 2: “Medium”; 1: “Slower”; 0: “Slow”
Distance	4: “Far”; 3: “Further”; 2: “Medium”; 1: “Closer”; 0: “Near”

**Table 2:** An example of a data instance from the dataset.

Node	Value	Description
altitude	4	The target altitude is “High altitude”.
time	3	The target time to arrival is “Relatively long”.
detected	1	Electromagnetic radiation from the target has been detected.
distance	4	The target distance is “Far”.
duration	2	The target electromagnetic detection time is “Relatively long detection time”.
heading	2	The target orientation is “Large”.
interval	2	The target time interval is “Long”.
latitude	0	The target latitude is “Small”.
longitude	3	The target longitude is “Relatively large”.
sensor	0	The target is detected by an electronic support/measurement system.
sources	1	The target is an “Other” type of Radiation Source.
speed	0	The target speed is “Slow”.
state	2	The target activity status is “Non-RTB”.
type	1	The target type is “Stealth carrier-based attack aircraft”.
threaten	2	The threat level of the target to us is “medium threat”.

**Table 3:** The sensitivity analysis results of each threat assessment model to the Threaten node based on the variance reduction indicator.

NB		TAN		Hillclimbing Search		Tree Search	
Node	$V_{r}$	Node	$V_{r}$	Node	$V_{r}$	Node	$V_{r}$
type	0.452339	type	0.452339	type	0.452339	type	0.452339
state	0.373774	state	0.373774	state	0.373774	state	0.373774
heading	0.234806	heading	0.234806	distance	0.239502	distance	0.246874
sensor	0.104329	sensor	0.104329	time	0.234805	heading	0.234806
interval	0.09527	interval	0.09527	heading	0.229909	time	0.180547
sources	0.050549	sources	0.050549	longitude	0.191044	longitude	0.127475
duration	0.039677	duration	0.039677	speed	0.178489	latitude	0.121023
detected	0.036223	detected	0.036223	latitude	0.142918	speed	0.120442
altitude	0.033699	altitude	0.033699	altitude	0.107484	sensor	0.104329
time	0	time	0	sensor	0.10161	interval	0.07746
speed	0	speed	0	interval	0.05856	altitude	0.062925
longitude	0	longitude	0	sources	0.050718	sources	0.050549
latitude	0	latitude	0	duration	0.036898	duration	0.039677
distance	0	distance	0	detected	0.036304	detected	0.036223

**Table 4:** The sensitivity analysis results of each threat assessment model to the Threaten node based on the mutual information indicator.

NB		TAN		Hillclimbing Search		Tree Search	
Node	MI	Node	MI	Node	MI	Node	MI
type	0.8431	type	0.8431	type	0.8431	type	0.8431
state	0.5062	state	0.5062	state	0.5062	state	0.5062
sensor	0.277	sensor	0.277	distance	0.4103	time	0.4676
heading	0.2739	heading	0.2739	time	0.391	distance	0.3506
sources	0.1901	sources	0.1901	speed	0.3152	speed	0.2985
duration	0.1705	duration	0.1705	heading	0.2685	sensor	0.277
interval	0.167	interval	0.167	longitude	0.2683	heading	0.2739
detected	0.164	detected	0.164	sensor	0.2665	longitude	0.2584
altitude	0.0599	altitude	0.0599	altitude	0.2618	sources	0.1901
time	0	time	0	latitude	0.2002	duration	0.1705
distance	0	distance	0	sources	0.1902	detected	0.164
latitude	0	latitude	0	duration	0.1645	altitude	0.1622
longitude	0	longitude	0	detected	0.1638	interval	0.1483
speed	0	speed	0	interval	0.0974	latitude	0.1468

**Table 5:** The sensitivity analysis results of each threat assessment model to the Threaten node based on the expected variance indicator.

NB		TAN		Hillclimbing Search		Tree Search	
Node	$V_{E}$	Node	$V_{E}$	Node	$V_{E}$	Node	$V_{E}$
state	1.330473	state	1.330473	state	1.330473	state	1.330473
type	1.122372	type	1.122372	type	1.122372	type	1.122372
sources	0.53004	sources	0.53004	sources	0.5269	sources	0.53004
heading	0.401594	heading	0.401594	heading	0.399965	heading	0.401594
sensor	0.107022	sensor	0.107022	distance	0.230282	time	0.235428
interval	0.103659	interval	0.103659	time	0.210783	distance	0.186231
duration	0.06478	duration	0.06478	speed	0.167205	speed	0.150398
detected	0.057179	detected	0.057179	longitude	0.166121	longitude	0.149314
altitude	0.030958	altitude	0.030958	latitude	0.139369	sensor	0.107022
time	0	time	0	altitude	0.107481	interval	0.100002
latitude	0	latitude	0	sensor	0.100354	latitude	0.091211
longitude	0	longitude	0	detected	0.057229	altitude	0.08969
speed	0	speed	0	interval	0.056346	duration	0.06478
distance	0	distance	0	duration	0.051926	detected	0.057179

**Table 6:** Conditional probability reasoning results of threat level nodes under different threat assessment models for a given scenario.

Node Value/Model	NB	TAN	Hillclimbing Search	Tree Search	Tree–Hillclimb Search
Threaten = 0	0.47	0.44	0.03	0.64	0.16
Threaten = 1	99.1	99.1	99.9	99.0	99.7
Threaten = 2	0.08	0.06	0.05	0.10	0.04
Threaten = 3	0.35	0.40	0.02	0.26	0.10

**Table 7:** Parameter complexity of each threat assessment model.

Model	NB	TAN	Hillclimbing Search	Tree Search	Tree–Hillclimb Search
Parameter Complexity	307	391	2040	222	403

**Table 8:** Processing time ^1^ of each threat assessment model.

Model	NB	TAN	Hillclimbing Search	Tree Search	Tree–Hillclimb Search
Structure Learning Time	0.0009	0.0010	1.0007	4.5323	0.0011
Parameter Learning Time	0.0359	0.0396	0.0432	0.0410	0.0366
Inference Time	0.0010	0.0005	0.0010	0.0010	0.0001

^1^ The unit of time is seconds.

**Table 9:** Conditional probability reasoning results of the threat level node after changing the precision of Distance.

Node Value/Model	NB	TAN	Hillclimbing Search	Tree Search	Tree–Hillclimb Search
threaten = 0	1.35	1.26	0.83	0.72	0.56
threaten = 1	97.7	97.6	98.4	98.0	98.3
threaten = 2	0.19	0.17	0.22	0.62	0.66
threaten = 3	0.61	0.97	0.55	0.66	0.48

**Table 10:** Conditional probability reasoning results of threat level node after changing the precision of Altitude.

Node Value/Model	NB	TAN	Hillclimbing Search	Tree Search	Tree–Hillclimb Search
threaten = 0	0.49	0.46	0.1	0.53	0.19
threaten = 1	99.1	99.0	99.6	99.0	99.4
threaten = 2	0.05	0.12	0.09	0.21	0.11
threaten = 3	0.36	0.42	0.21	0.26	0.30

**Table 11:** Results of the ablation study on expert constraints.

Model	Accuracy (%)	Parameter Complexity	Average Inference Time (s)
Tree–Hillclimb Search model	99.7	403	0.0001
Hillclimbing Search model	99.9	2040	0.0010
Expert experience model	99.1	307	0.0010

## Data Availability

Data are contained within the article.

## Abbreviations

The following abbreviations are used in this manuscript:

AHP	Analytic Hierarchy Process
BDe	Bayesian Dirichlet equivalent
BDeu	Bayesian Dirichlet equivalent uniform
BFS	Breadth-First Search
BIC	Bayesian Information Criterion
BNPL	Bayesian networks parameter learning
BNs	Bayesian networks
BNSL	Bayesian networks structure learning
CPDAG	complete partial directed acyclic graph
CPDs	Conditional Probability Distributions
CPTs	Conditional Probability Tables
DAG	directed acyclic graph
MADM	Multi-Attribute Decision-Making
MDL	Minimum Description Length
MMPC	Max-Min Parents and Children
MWST	Maximum Weight Spanning Tree
NB	Naive Bayes model
OODA	Observe, Orient, Decide, Act
PC	Peter–Clark algorithm
PDAG	partial directed acyclic graph
SSA	Salp Swarm Algorithm
SVMs	support vector machines
TAN	Tree Augmented Naive Bayes model
TOPSIS	Technique for Order Preference by Similarity to Ideal Solution
